# Performance stability despite iteration: evaluating DeepSeek and ChatGPT on Chinese medical licensing examinations

**DOI:** 10.3389/fmed.2026.1874194

**Published:** 2026-06-24

**Authors:** Zhiheng Wang, Yifan Qin, Jin Wu

**Affiliations:** Department of Anesthesiology, Affiliated Hospital of Jiangsu University, Zhenjiang, China

**Keywords:** ChatGPT, Chinese National Medical Licensing Examination, DeepSeek, error analysis, large language models, longitudinal evaluation

## Abstract

**Background:**

Large Language Models (LLMs) hold substantial potential in medical education. In our previous work, we evaluated the performance of DeepSeek-R1 and ChatGPT-4o on the Chinese National Medical Licensing Examination (CNMLE). Following performance upgrades in December 2025, DeepSeek-V3.2 and ChatGPT-5.2 were released. This study aimed to longitudinally assess the performance evolution of DeepSeek (R1 vs. V3.2) and ChatGPT (4o vs. 5.2) using the 2024 CNMLE as a baseline and to explore their performance on the 2025 CNMLE.

**Methods:**

We tested DeepSeek-V3.2 and ChatGPT-5.2 on 600 multiple-choice questions from the written part of the 2024 CNMLE, and compared the results with historical data from DeepSeek-R1 and ChatGPT-4o. The questions consisted of 4 units and were divided into low-difficulty and high-difficulty groups according to different difficulty levels. Additionally, the two latest LLMs were assessed on 600 questions from the written part of the 2025 CNMLE.

**Results:**

In the 2024 CNMLE, overall accuracy for the DeepSeek series (R1 vs. V3.2: 92.0% vs. 91.0%) and ChatGPT series (4o vs. 5.2: 87.2% vs. 89.3%) showed no significant differences (all *p* > 0.05). The accuracy gap between the two series narrowed from 4.8% (DeepSeek-R1 vs. ChatGPT-4o, *p* < 0.05) to 1.7% (DeepSeek-V3.2 vs. ChatGPT-5.2, *p* = 0.332). Subgroup analyses by unit and difficulty revealed no statistically significant differences, either in longitudinal comparisons between successive versions within each series or in cross-sectional comparisons between the latest versions of the two series. On the 2025 CNMLE, DeepSeek-V3.2 achieved significantly higher overall accuracy than ChatGPT-5.2 (94.7% vs. 89.3%, *p* < 0.05), with superior performance in Unit 2 and in both the low- and high-difficulty groups (*p* < 0.05). Error analysis showed no significant differences across model versions in error type classification or clinical risk rating, despite a substantial proportion of high-risk clinical errors.

**Conclusion:**

Benchmarked against the 2024 CNMLE, iterative updates did not yield significant performance gains for either LLM series. However, DeepSeek-V3.2 demonstrated a performance advantage on the 2025 CNMLE.

## Background

Large language models (LLMs) are increasingly integrated into people’s daily lives, demonstrating promising application prospects (1). In the medical field, LLMs have been widely applied across various domains, such as clinical decision support (2), medical education (3), and medical research (4), with their powerful natural language processing capabilities offering new possibilities for the intelligent development of medical practice (5).

In the domain of medical education, the Medical Licensing Examination (MLE) serves as a core tool for assessing the knowledge base of medical students and clinical practitioners (6). In recent years, numerous studies have evaluated the performance of LLMs on various national medical examinations, and the findings can be summarized into three main observations. 1. Iterative version updates of LLMs bring tangible performance improvements. Bicknell et al. (7) found that GPT-4o achieved a significantly higher accuracy (90.4%) on the United States Medical Licensing Examination (USMLE) compared to GPT-4 (81.1%), GPT-3.5 (60.0%), and the average level of medical students (59.3%). Luo et al. (8) confirmed on the 2020 and 2021 Chinese National Medical Licensing Examination (CNMLE) that GPT-4o significantly outperformed GPT-4 and GPT-3.5 (*p* < 0.001). Sheikhalishahi et al. (9) further showed that ChatGPT-5 (74.5%) continued to improve over ChatGPT-4o (68.9%) on the 2025 Iranian Internal Medicine Subspecialty Board Examination. A systematic review by Liu et al. (10) (45 studies) reported that GPT-4 achieved an overall accuracy of 81%, significantly higher than GPT-3.5’s 58%. 2. LLM performance is influenced by question difficulty and medical specialty domain. Liu et al. (11) demonstrated on the Japanese Medical Licensing Examination (JMLE) that question difficulty significantly affects model accuracy, and that model performance across different specialties is positively correlated with the volume of relevant publications. 3. Domestic Chinese LLMs perform better on the CNMLE. Wang et al. (12) evaluated seven models on the 2021 CNMLE and showed that DeepSeek-R1 (96%) and DeepSeek-V3 (93%) both significantly outperformed GPT-o1 pro (75%), GPT-o3 mini (75.8%), and GPT-4o (75.3%) (all *p* < 0.001).

Our previous comparative study demonstrated that both DeepSeek-R1, a domestically developed Chinese LLM, and ChatGPT-4o, a mainstream Western LLM, exhibited excellent performance on the 2024 CNMLE, with scores substantially exceeding the average scores of human examinees. Between the two models, DeepSeek-R1 exhibited superior performance, suggesting stronger potential for application in the field of medical education in China (13). As a free and open-source Chinese LLM, DeepSeek-R1 leverages reinforcement learning and mixture of experts technologies to balance performance and cost-effectiveness when handling complex logical reasoning tasks (14). In contrast, ChatGPT-4o is a paid and closed-source multimodal LLM. With its extensive pre-training corpus and large-scale supervised fine-tuning techniques, it excels in daily conversations and tasks requiring creative generation (15).

In the rapidly advancing era of artificial intelligence, with the integration of new technologies and the deployment of increasingly large-scale computational resources, both DeepSeek and OpenAI have released updated versions of their LLM products, namely DeepSeek-V3.2 and ChatGPT-5.2, which are claimed to possess enhanced reasoning and knowledge capabilities. Released on December 1, 2025, DeepSeek-V3.2 employs its proprietary DeepSeek Sparse Attention (DSA) technology, achieving GPT-5-level performance across multiple reasoning benchmarks while reducing long-text inference costs by more than 60%, thereby achieving an optimal balance between high performance and computational efficiency (16). ChatGPT-5.2, released on December 11, 2025, adopts a novel model architecture, reaching or exceeding human expert-level performance in 70.9% of tasks on the GDPval benchmark while reducing the model hallucination rate by approximately 38%, representing a comprehensive advancement in professional knowledge capabilities (17). These developments raise two critical questions: when evaluated on the same fixed set of examination questions (the 2024 CNMLE), do these iterative updates represent genuine progress in the core medical knowledge of LLMs? This question pertains directly to the real benefits of model iteration, specifically whether such iteration merely improves the models’ ability to “perform” on previously seen questions, or whether it corresponds to substantive improvement in core medical knowledge. Additionally, how do these latest models perform when faced with entirely new examination content (the 2025 CNMLE)?

This study aimed to use the 2024 CNMLE as a benchmark to longitudinally compare the DeepSeek series (R1 vs. V3.2) and the ChatGPT series (4o vs. 5.2) in terms of overall accuracy, unit-specific accuracy, and difficulty-level accuracy, to conduct cross-sectional comparisons between the latest model versions, and to further evaluate the performance of the two latest models on the 2025 CNMLE, while additionally performing error type classification and clinical risk rating of incorrect answers.

## Methods

### Source of questions

The CNMLE is a national-level licensing examination for the medical profession in China, designed to assess whether medical graduates possess the fundamental medical knowledge, clinical skills, and professional competencies required for clinical practice. It serves as the gold standard for evaluating a physician’s practice capability. The examination consists of two parts: a practical skills assessment and a written test. The written test comprises a total of 600 questions, each worth one point, with a passing score set at 360 points or above (18).

#### 2024 CNMLE questions

The 600 multiple-choice questions from the written part of the CNMLE administered in August 2024 were selected as the testing material. All questions were text-based, without images or tables, and were included in their original form without deletions or modifications. The questions and corresponding reference answers were provided by the Yikaobang application, developed by Beijing Medical Examination Assistance Technology Co., Ltd., and the reference answers were verified by two senior clinicians. The examination questions comprised 4 units. Unit 1 was designed to assess candidates’ mastery of foundational medical knowledge, while Units 2 to 4 focused on evaluating practical abilities in clinical reasoning and diagnostic decision-making. Unit 1 covered basic medical knowledge, preventive medicine, and health regulations; Unit 2 involved the cardiovascular, urological, musculoskeletal, and endocrine systems; Unit 3 examined the digestive, respiratory, and other related systems; and Unit 4 focused on the female reproductive system, pediatric diseases, and the neuropsychiatric system. Each unit contained 150 questions, including the following question types: A1 type (single sentence best choice question), A2 type (case summary best choice question), A3/A4 type (case group best choice question), and B1 type (standard matching question) (19).

#### 2025 CNMLE questions

The 600 multiple-choice questions from the written part of the CNMLE administered in August 2025 were selected as the testing material. The structure of the examination was identical to that of the 2024 CNMLE, with all questions being text-based. The questions, along with the reference answers that had been verified by two clinicians with senior professional titles, were also provided by the Yikaobang application.

### Difficulty stratification

Based on the difficulty rating of each question provided by the Yikaobang application (derived from the accuracy rates of real users), the questions were divided into low-difficulty and high-difficulty groups. The difficulty rating used a one-to-five-star system, with a higher number of stars indicating greater difficulty. Questions rated 1 to 3 stars were classified into the low-difficulty group, and those rated 4 to 5 stars were classified into the high-difficulty group. In the 2024 CNMLE, there were 386 questions in the low-difficulty group and 214 in the high-difficulty group; in the 2025 CNMLE, there were 342 questions in the low-difficulty group and 258 in the high-difficulty group.

### Models and testing environment

#### Model versions

The models tested in this study included DeepSeek-V3.2 (released on December 1, 2025, with a knowledge base cutoff of July 2024) and ChatGPT-5.2 (released on December 11, 2025, with a knowledge base cutoff of August 2025). The release dates and knowledge base cutoffs for DeepSeek-R1 and ChatGPT-4o are detailed in our previous study (13).

#### Testing timeline

Testing for this study was conducted from February 4 to February 16, 2026. DeepSeek-V3.2 completed the 2024 and 2025 examination questions between February 4 and February 10, while ChatGPT-5.2 completed the same testing between February 11 and February 16. The testing of DeepSeek-R1 and ChatGPT-4o was carried out from March 3 to March 9, 2025.

#### Testing setup

All questions were input in Chinese into the official web-based chat interfaces of DeepSeek-V3.2 (free version) and ChatGPT-5.2 (paid version). To ensure that model responses were based solely on their internal knowledge bases, the “Online Search” function was disabled for all queries. For DeepSeek-V3.2, the “Deep Thinking” mode was enabled to record its reasoning process. Since certain queries could still trigger online searches when the “Intelligent Search” option was disabled, we explicitly instructed the model not to search the internet for answers to avoid any influence from external information. For ChatGPT-5.2, the “Thinking” mode was enabled to enhance its reasoning capabilities, and a similar explicit instruction “do not search the internet for answers” was given to prevent data contamination. Each question was submitted only once. The final answer provided by the model was recorded and compared against the reference answer.

### Error analysis

Following methods used in previous studies (20, 21), we performed error type classification and clinical risk rating for the incorrect answers generated by the LLMs.

#### Error type classification

Incorrect answers were categorized into four types: (1) logical errors (errors in reasoning processes or misjudgment of causal relationships); (2) informational errors (knowledge gaps, outdated knowledge, or memory inaccuracies); (3) statistical errors (errors in probability judgment or numerical calculation); and (4) ambiguous errors (misinterpretation of the question or instruction-execution errors).

#### Clinical risk rating

Two senior clinicians independently rated the clinical risk of each incorrect answer, classifying errors as either high-risk or low-risk. A high-risk clinical error was defined as an error that could directly affect patient safety or treatment outcomes, including: (1) diagnostic errors; (2) inappropriate sequencing of treatments or examinations; (3) misjudgment of critical clinical information (e.g., contraindications, allergy history); (4) errors in emergency management; and (5) errors involving high-risk populations such as children, pregnant women, and elderly patients. A low-risk clinical error was defined as an error that did not affect patient safety or treatment outcomes. Discrepancies between the two clinicians were resolved through consensus discussion.

### Outcome measures

#### Primary outcome measure

The primary outcome measure was the change in overall accuracy between the DeepSeek series (R1 vs. V3.2) and between the ChatGPT series (4o vs. 5.2) on the 2024 CNMLE.

#### Secondary outcome measures

The secondary outcome measures included: (1) differences in unit-specific accuracy and difficulty-stratified accuracy between the DeepSeek series (R1 vs. V3.2) and between the ChatGPT series (4o vs. 5.2) on the 2024 CNMLE; (2) differences in overall accuracy, unit-specific accuracy, and difficulty-stratified accuracy between DeepSeek-V3.2 and ChatGPT-5.2 on the 2024 CNMLE; (3) differences in overall accuracy, unit-specific accuracy, and difficulty-stratified accuracy between DeepSeek-V3.2 and ChatGPT-5.2 on the 2025 CNMLE; and (4) error type analysis and clinical risk rating of incorrect answers for both model series on the 2024 and 2025 CNMLE.

### Statistical analysis

Statistical analyses were performed using SPSS 27.0 software. Comparisons of overall accuracy, unit-specific accuracy, and difficulty-stratified accuracy between models were conducted using the chi-square test or Fisher’s exact test when expected frequencies were less than 5. Differences in error type classification and clinical risk ratings were also examined using the aforementioned methods. Inter-rater agreement for error type classification and clinical risk rating was assessed using Cohen’s kappa (*κ*). The significance level was set at *α* = 0.05, with *p* < 0.05 considered statistically significant.

## Results

### Performance of LLMs on the 2024 CNMLE

Data on overall accuracy, unit-specific accuracy, and difficulty-stratified accuracy for DeepSeek-R1 and ChatGPT-4o on the 2024 CNMLE were obtained from our previous study (13).

**Figure 1:**
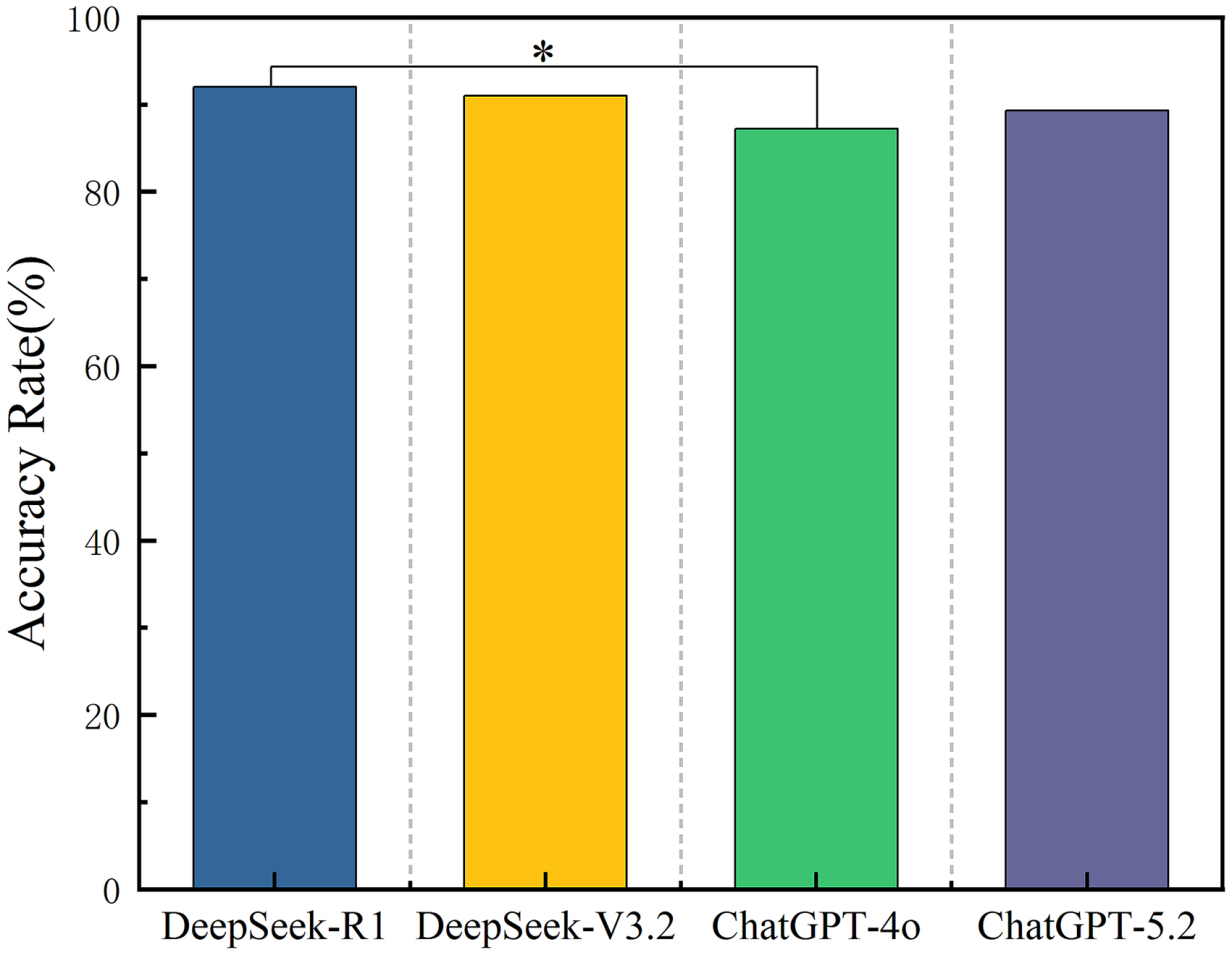
Overall accuracy of the four models on the 2024 CNMLE (DeepSeek-R1: 92.0%; DeepSeek-V3.2: 91.0%; ChatGPT-4o: 87.2%; ChatGPT-5.2: 89.3%). The asterisk indicates a significant difference (DeepSeek-R1 vs. ChatGPT-4o, *p* = 0.006). Other pairwise comparisons were not statistically significant.

Among the 600 questions of the 2024 CNMLE, no significant difference in overall accuracy was observed before and after iteration for the DeepSeek series: DeepSeek-R1 achieved an accuracy of 92.0% (552/600), while DeepSeek-V3.2 achieved 91.0% (546/600), with no statistically significant difference (*p* = 0.535). Similarly, for the ChatGPT series, overall accuracy remained stable before and after iteration: ChatGPT-4o achieved an accuracy of 87.2% (523/600), while ChatGPT-5.2 achieved 89.3% (536/600), with no statistically significant difference (*p* = 0.244). The overall accuracies of the four models on the 2024 CNMLE are shown in Figure 1.

Cross-sectional comparisons revealed a narrowing of the accuracy gap between the two LLM series following model iteration. In the comparison of earlier versions, DeepSeek-R1 significantly outperformed ChatGPT-4o (92.0% vs. 87.2%), with a difference of 4.8% (*p* = 0.006). In the comparison of the updated versions, DeepSeek-V3.2 and ChatGPT-5.2 achieved overall accuracies of 91.0 and 89.3%, respectively, reducing the gap to 1.7%, though this difference was not statistically significant (*p* = 0.332).

Further analyses across the 4 units and the low- and high-difficulty groups showed that no subgroup exhibited statistically significant differences in accuracy (all *p* > 0.05), whether for longitudinal comparisons within the DeepSeek series (R1 vs. V3.2) or the ChatGPT series (4o vs. 5.2), or for cross-sectional comparisons between the updated models (V3.2 vs. 5.2). Notably, both the DeepSeek and ChatGPT series exhibited a trend of decreasing accuracy with increasing question difficulty in both old and new versions, and this trend remained consistent before and after model iteration. Detailed results are presented in Tables 1–3.

**Table 1:** Comparison of accuracy [*n* (%)] between DeepSeek-R1 and DeepSeek-V3.2 on the 2024 CNMLE by unit and difficulty group.

Model	Unit 1	Unit 2	Unit 3	Unit 4	Low-difficulty group	High-difficulty group
DeepSeek-R1	136 (90.7%)	139 (92.7%)	141 (94.0%)	136 (90.7%)	370 (95.9%)	182 (85.0%)
DeepSeek-V3.2	135 (90.0%)	136 (90.7%)	142 (94.7%)	133 (88.7%)	365 (94.6%)	181 (84.6%)
*p* value	0.845	0.531	0.803	0.569	0.400	0.893

Each unit comprised 150 questions; the low-difficulty group included 386 questions and the high-difficulty group 214 questions.

**Table 2:** Comparison of accuracy [*n* (%)] between ChatGPT-4o and ChatGPT-5.2 on the 2024 CNMLE by unit and difficulty group.

Model	Unit 1	Unit 2	Unit 3	Unit 4	Low-difficulty group	High-difficulty group
ChatGPT-4o	126 (84.0%)	131 (87.3%)	135 (90.0%)	131 (87.3%)	355 (92.0%)	168 (78.5%)
ChatGPT-5.2	131 (87.3%)	135 (90.0%)	138 (92.0%)	132 (88.0%)	359 (93.0%)	177 (82.7%)
*P* value	0.410	0.466	0.545	0.861	0.585	0.271

Each unit comprised 150 questions; the low-difficulty group included 386 questions and the high-difficulty group 214 questions.

**Table 3:** Comparison of accuracy [*n* (%)] between DeepSeek-V3.2 and ChatGPT-5.2 on the 2024 CNMLE by unit and difficulty group.

Model	Unit 1	Unit 2	Unit 3	Unit 4	Low-difficulty group	High-difficulty group
DeepSeek-V3.2	135 (90.0%)	136 (90.7%)	142 (94.7%)	133 (88.7%)	365 (94.6%)	181 (84.6%)
ChatGPT-5.2	131 (87.3%)	135 (90.0%)	138 (92.0%)	132 (88.0%)	359 (93.0%)	177 (82.7%)
*P* value	0.466	0.845	0.355	0.857	0.371	0.601

Each unit comprised 150 questions; the low-difficulty group included 386 questions and the high-difficulty group 214 questions.

### Performance of LLMs on the 2025 CNMLE

Among the 600 questions of the 2025 CNMLE, DeepSeek-V3.2 achieved an overall accuracy of 94.7% (568/600), which was significantly higher than that of ChatGPT-5.2 (89.3%, 536/600), with a difference of 5.4% (*p* < 0.001).

Subgroup analyses indicated that DeepSeek-V3.2 significantly outperformed ChatGPT-5.2 in Unit 2 (the cardiovascular, urinary, musculoskeletal, and endocrine systems), as well as in both the low- and high-difficulty groups (*p* < 0.05). In the remaining 3 units (Units 1, 3, and 4), DeepSeek-V3.2 outperformed ChatGPT-5.2 in terms of the number of correct answers, although the differences were not statistically significant (*p* > 0.05). Detailed results are presented in Table 4.

**Table 4:** Comparison of accuracy [*n* (%)] between DeepSeek-V3.2 and ChatGPT-5.2 on the 2025 CNMLE by unit and difficulty group.

Model	Unit 1	Unit 2	Unit 3	Unit 4	Low-difficulty group	High-difficulty group
DeepSeek-V3.2	139 (92.7%)	147 (98.0%)	137 (91.3%)	145 (96.7%)	335 (98.0%)	233 (90.3%)
ChatGPT-5.2	133 (88.7%)	130 (86.7%)	130 (86.7%)	143 (95.3%)	325 (95.0%)	211 (81.8%)
*P* value	0.234	<0.001	0.196	0.556	0.038	0.005

Each unit comprised 150 questions; the low-difficulty group included 342 questions and the high-difficulty group 258 questions.

### Error analysis

#### 2024 CNMLE

No significant differences were observed in error type classification, whether in the longitudinal comparisons within the DeepSeek series (R1 vs. V3.2), within the ChatGPT series (4o vs. 5.2), or in the cross-sectional comparisons between the two versions (R1 vs. 4o, V3.2 vs. 5.2) (all *p* > 0.05). For all four models, informational errors predominated (47.9–67.5%), followed by logical errors (23.4–37.5%), with statistical errors and ambiguous errors accounting for smaller proportions. Regarding clinical risk rating, the proportion of high-risk clinical errors among incorrect answers for each model ranged from 59.3 to 65.6%, with no statistically significant differences (all *p* > 0.05).

#### 2025 CNMLE

There was no significant difference in error type classification between DeepSeek-V3.2 and ChatGPT-5.2 (*p* > 0.05). For DeepSeek-V3.2, informational errors were the most common (75.0%), followed by logical errors (25.0%), with no statistical or ambiguous errors observed. For ChatGPT-5.2, informational errors also predominated (73.4%), followed by logical errors (23.4%) and ambiguous errors (3.1%), with no statistical errors observed. In terms of clinical risk rating, the proportion of high-risk clinical errors was 46.9% for DeepSeek-V3.2 and 59.4% for ChatGPT-5.2, with no statistically significant difference (*p* > 0.05). Cohen’s kappa for error type classification was 0.918 (*p* < 0.05), and for clinical risk rating was 0.925 (*p* < 0.05), indicating almost perfect inter-rater agreement. Detailed error analysis results are presented in Table 5.

**Table 5:** Error analysis of two LLM series on the 2024 and 2025 CNMLE [*n* (%)].

Error type	DeepSeek-R1 (2024)	ChatGPT-4o (2024)	DeepSeek-V3.2 (2024)	ChatGPT-5.2 (2024)	DeepSeek-V3.2 (2025)	ChatGPT-5.2 (2025)
Total errors	48	77	54	64	32	64
Logical errors	18 (37.5%)	18 (23.4%)	20 (37.0%)	24 (37.5%)	8 (25.0%)	15 (23.4%)
Informational errors	23 (47.9%)	52 (67.5%)	27 (50%)	33 (51.6%)	24 (75.0%)	47 (73.4%)
Statistical errors	1 (2.1%)	2 (2.6%)	1 (1.9%)	1 (1.6%)	0 (0.0%)	0 (0.0%)
Ambiguous errors	6 (12.5%)	5 (6.5%)	6 (11.1%)	6 (9.4%)	0 (0.0%)	2 (3.1%)
High-risk clinical errors	31 (64.6%)	50 (64.9%)	32 (59.3%)	42 (65.6%)	15 (46.9%)	38 (59.4%)
Low-risk clinical errors	17 (35.4%)	27 (35.1%)	22 (40.7%)	22 (34.4%)	17 (53.1%)	26 (40.6%)

## Discussion

This study evaluated the performance of DeepSeek-V3.2 and ChatGPT-5.2 on the written part of the 2024 and 2025 CNMLE. Combined with the test data from the previous-generation models on the 2024 CNMLE from our prior study, we longitudinally compared the specific changes between the two generations of the DeepSeek series and the ChatGPT series, and further analyzed the incorrect answers. The results indicated that, on the 2024 CNMLE, the updated models did not exhibit significant improvements over their respective predecessors, with performance remaining largely stable. On the 2025 CNMLE, DeepSeek-V3.2 significantly outperformed ChatGPT-5.2 in overall accuracy, Unit 2, and both the low- and high-difficulty groups. Error analyses revealed that incorrect responses were predominantly informational and logical, accompanied by a substantial proportion of high-risk clinical errors.

On the 2024 CNMLE, no significant performance changes were observed after iteration for either model series. The overall accuracy of ChatGPT-5.2 increased by only approximately 2% compared with ChatGPT-4o, whereas DeepSeek-V3.2 even showed a 1% decrease in overall accuracy relative to DeepSeek-R1. This contrasts sharply with the substantial improvements reported for ChatGPT-3.5 to ChatGPT-4.0 on the 2022 CNMLE (22, 23). Two factors may account for this observation. On the one hand, the previous-generation models had already achieved outstanding performance on the 2024 CNMLE, with accuracy far exceeding the average level of actual test takers that year, leaving limited room for further improvement (13). This suggests that LLMs, after undergoing rapid iteration during their early development, have entered a relatively mature stage, with diminishing marginal gains in their clinical medical knowledge bases. On the other hand, the development of the new-generation models has focused more on reducing inference costs and improving reasoning efficiency (24), rather than on broader coverage of medical knowledge. DeepSeek-V3.2 employs its proprietary DSA technology, which incorporates two key screening mechanisms: the Lightning Indexer and the Fine-grained Token Selection module. These mechanisms effectively address the efficiency and cost bottlenecks encountered by LLMs when processing long texts. The use of DSA enables DeepSeek-V3.2 to substantially reduce computational complexity when handling long texts without sacrificing model performance, thereby improving computational efficiency while reducing inference overhead (25). However, this “coarse-to-fine” attention mechanism of DSA may affect the retrieval of certain detailed medical knowledge, potentially contributing to the slight decline in overall accuracy.

DeepSeek-V3.2 demonstrated superior performance on the written part of the 2025 CNMLE compared with ChatGPT-5.2, continuing the performance advantage previously observed for DeepSeek-R1 over ChatGPT-4o on the 2024 CNMLE. Unlike prior studies, DeepSeek-V3.2 not only showed advantages in overall accuracy and the low-difficulty group but also performed better in Unit 2 and the high-difficulty group. This may be attributed to the following two factors. On the one hand, the overall difficulty of the 2025 CNMLE increased compared with previous years, incorporating the latest medical guidelines while placing greater emphasis on clinical practice. Many questions involved clinical scenarios, including medication usage not explicitly covered in textbooks, with longer textual content and higher demands for keyword extraction (26). Unit 2 primarily covers the cardiovascular, urological, musculoskeletal, and endocrine systems, all of which are core clinical systems, and includes numerous questions that closely reflect real-world clinical practice and involve substantial textual content. As a domestically developed Chinese LLM, DeepSeek-V3.2 had greater opportunities than the Western LLM ChatGPT-5.2 to learn the latest Chinese medical guidelines and clinical application knowledge during its pre-training phase, and it possesses stronger capabilities in processing the Chinese language. On the other hand, similar to DeepSeek-R1, DeepSeek-V3.2 retains a detailed reasoning process when the “Deep Thinking” mode is enabled, reflecting the application of chain-of-thought technology, which enhances the credibility of model responses while improving its ability to solve complex clinical scenarios (27). In contrast, the “Thinking” mode of ChatGPT-5.2 does not display its reasoning process. Additionally, the application of a Scalable Reinforcement Learning Framework in DeepSeek-V3.2 allowed unprecedented computational investment during the post-training phase, substantially enhancing the model’s capacity to solve complex reasoning problems (16). Consequently, DeepSeek-V3.2 achieved a marked advantage when addressing high-difficulty questions involving extensive and complex clinical reasoning.

Regarding error analysis, iteration between old and new model versions did not alter the predominant error types, which remained primarily informational and logical errors, and a high proportion of high-risk clinical errors persisted among the incorrect answers, consistent with findings from previous studies (20). Even for DeepSeek-V3.2 on the 2025 CNMLE, which had the fewest overall errors, 46.9% of incorrect responses were classified as high clinical risk and could potentially pose a direct threat to patient safety and treatment outcomes. These results underscore the need for caution when applying LLMs in real-world clinical practice.

This study has several limitations. First, because DeepSeek-R1 was no longer accessible via the official website at the time of testing, we were only able to compare iteration differences between the two LLM series on the 2024 CNMLE. Consequently, we could not evaluate the performance of the older-version models, DeepSeek-R1 and ChatGPT-4o, on the 2025 CNMLE, which would have allowed a more comprehensive comparison between old and new versions. Second, as ChatGPT-5.2 does not display reasoning time, this study was unable to perform comparative analyses of LLM reasoning time and therefore could not fully capture the efficiency change conferred by the DSA technology in DeepSeek-V3.2. Third, given the rapid development of LLMs, the results reported here reflect model performance only at the time of testing, and future iterations may yield different outcomes. Fourth, this study only assessed LLM performance on closed-ended multiple-choice questions and did not include open-ended questions, which limits the comprehensiveness of our evaluation of performance changes. Fifth, to better compare the changes between the older and newer models on the 2024 CNMLE, we adhered to the methodology of previous studies and did not incorporate Chinese-English comparisons or diverse prompting strategies. Sixth, Because ChatGPT-5.2 has a knowledge cutoff of August 2025, data contamination is possible when testing on the 2024 CNMLE. DeepSeek-V3.2 has a knowledge cutoff of July 2024, which is theoretically before the examination, but we cannot completely rule out the possibility that the questions were accessed through other channels (e.g., post-training data collection). Finally, different question types (A1, A2, A3/A4, B1) may pose varying challenges in reasoning and information retrieval; future studies should stratify by question type to gain a deeper understanding of model capabilities.

## Conclusion

This study indicates that, benchmarked against the 2024 CNMLE, iterative updates of the DeepSeek and ChatGPT series did not translate into measurable gains in core medical knowledge, suggesting that LLMs have entered a relatively mature stage on fixed examinations. DeepSeek-V3.2 maintained its advantage on the CNMLE; nevertheless, caution is warranted when applying it in medical education.

## Data Availability

The raw data supporting the conclusions of this article will be made available by the authors, without undue reservation.
